# Modelo participativo para el abordaje de la violencia contra las mujeres en La Araucanía, Chile

**DOI:** 10.26633/RPSP.2017.69

**Published:** 2017-04-28

**Authors:** Lucy Mirtha Ketterer Romero, Cecilia Mayorga Muñoz, Marcelo Carrasco Henríquez, Abel Soto Higueras, Ana Tragolaf Ancalaf, Luis Nitrihual, Carlos del Valle

**Affiliations:** 1 Universidad de La Frontera Trabajo Social Temuco Chile Universidad de La Frontera, Trabajo Social, Temuco, Chile.; 2 Observatorio equidad en salud según género y pueblo mapuche Temuco Chile Observatorio equidad en salud, según género y pueblo mapuche, Temuco, Chile.

**Keywords:** Violencia doméstica, investigación participativa basada en la comunidad, organización comunitaria, salud pública, Chile, Domestic violence, community-based participatory research, communitarian organization, public health, Chile, Violência doméstica, pesquisa participativa baseada na comunidade, organização comunitária, saúde pública, Chile

## Abstract

La violencia contra la mujer se considera un problema de salud pública que afecta a las mujeres en todo el mundo. Recientemente, el Consejo Directivo de la Organización Panamericana de la Salud declaró sus graves repercusiones sociales y económicas en la Región de las Américas y se comprometió a emprender acciones en los servicios de salud para afrontar el problema. En ese marco, se presentan los pasos de una investigación-acción-participativa (IAP), que se está desarrollando en el territorio wenteche de la región de La Araucanía de Chile, que apuesta por fortalecer los vínculos comunitarios, revalorizar los espacios de diálogo con y entre las personas del territorio, la participación social y la democracia en la generación de conocimientos pertinentes y participativos sobre este problema, y obtener información para diseñar un modelo de intervención que se adecúe a las características locales.

## Abstract (en)

Violence against women is considered a public health problem that affects women worldwide. Recently, the Directing Council of the Pan American Health Organization declared its serious socioeconomic impact in the Region of the Americas and committed to undertaking actions in the health services to address this problem. Within that framework, this paper describes the steps of a participatory action research (PAR) approach that is being implemented in the Wenteche Territory of the La Araucanía region of Chile, which aims to strengthen community bonds, rekindle opportunities for dialogue with and among the people of the territory, foster social participation and democracy in the generation of pertinent, participatory knowledge regarding this problem, and obtain information to support design of an intervention model adapted to local characteristics.

## RESUMO

A violência contra a mulher é um problema de saúde pública que atinge mulheres em todo o mundo. Recentemente, o Conselho Diretor da Organização Pan-Americana da Saúde reconheceu as sérias repercussões socioeconômicas do problema na Região das Américas e se comprometeu a realizar ações nos serviços de saúde para combatê-lo. Como parte deste enquadramento, são apresentadas as etapas de uma pesquisa-ação participativa sendo desenvolvida no território wenteche da região de La Araucanía, no Chile, que visa reforçar os vínculos na comunidade, revalorizar os espaços de diálogo com e entre os habitantes locais, a participação social e a democracia na geração de conhecimento pertinente e participativo sobre esta problemática e obter dados para a elaboração de um modelo de intervenção adequado às características locais.

En la actualidad, la violencia contra la mujer se considera un problema de salud púbica que afecta a las mujeres de todo el mundo. Investigaciones recientes señalan que entre 10 y 50% de las mujeres en el mundo ha sufrido sus efectos (1–3). Recientemente, el 54° Consejo Directivo de la Organización Panamericana de la Salud (OPS) declaró sus graves repercusiones sociales y económicas en la Región de las Américas y se comprometió a mejorar los servicios de salud para responder a este problema (4).

Estudios realizados por el Servicio Nacional de la Mujer en La Araucanía de Chile indican que 35% de mujeres de 15 a 49 años de edad que viven en esta zona ha padecido violencia psicológica, 19%, física y 11%, sexual, lo cual la sitúa entre las cinco regiones del país con mayores índices de denuncias (5), e impacta negativamente tanto en su salud física como en aspectos emocionales, económicos y de calidad de sus vidas (6).

Las políticas públicas implementadas por el Estado chileno para hacer frente a este problema se canalizan por dos vías. La primera es la de la judicialización, a través de la implantación de un marco legal sancionatorio. En Chile, la primera Ley de Violencia Intrafamiliar Número 19.325 data de 1994 (7), y en 2005 se aprobó la Ley No. 20.066, que está en vigor en la actualidad (8). La segunda es la de la medicalización, mediante la cual se ofrece tratamiento a las víctimas y eventualmente a sus familias. No obstante, poco se trabaja en la prevención de esta forma de relación social naturalizada entre hombres y mujeres y menos aún en la promoción de buenas prácticas basadas en valores como el respeto y el apoyo mutuo.

En el Observatorio de Equidad en Salud (http://observatoriogenerosalud.ufro.cl/) nos planteamos en 2013 investigar las experiencias en este tipo de violencia de hombres y mujeres del pueblo mapuche de La Araucanía en el territorio de Boyeco. Esta zona cubre un sector de comunidades mapuche, ubicadas a 12 kilómetros de la ciudad de Temuco, y forma parte del denominado territorio *wenteche*, en mapuzungun (idioma mapuche).

De este modo, surgió la Investigación Acción Participativa (IAP) denominada *Violencia de género y sus representaciones sociales en el territorio wenteche de la región de La Araucanía* cuyo objetivo general es conocer las representaciones sociales de la violencia de género en comunidades mapuche wenteche rurales con objeto de obtener información para diseñar un modelo de intervención que se adecúe a las características locales.

## MÉTODOS EMPLEADOS EN EL PROCESO

La investigación está sustentada en el paradigma sociocrítico y tiene como modelo metodológico la IAP, que [no] busca “... [ni] la transformación objetiva (separada de la subjetividad) ni la actividad subjetiva (separada de la objetividad), sino la unidad de ambos momentos... supone cierta relación mutua en virtud de la cual la praxis funda a la teoría, la nutre e impulsa a la vez que la teoría se integra como un momento necesario en ella...como crítica... como compromiso...como laboratorio... como conciencia...y como autocrítica...” (9). Esta forma de abordar el estudio de los problemas sociales permite, además de construir conocimientos colectivos, generar herramientas críticas para apoyar la solución de los problemas y beneficiar, así, tanto a las comunidades mapuche participantes como a los equipos profesionales de la salud y a otros actores sociales que trabajan en el territorio. Durante el diseño y el desarrollo de la investigación se han generado procesos de participación comunitaria que promocionan la participación y la toma de decisiones de los actores afectados de un modo u otro por el problema de la violencia contra las mujeres. A continuación, se describen los pasos metodológicos desarrollados hasta la fecha en esta investigación, que conforman un modelo comunitario de abordaje de este tipo de violencia.

### Primer paso: primeros contactos, evaluación del problema, diseño participativo de la investigación y acciones en el territorio

En esta primera etapa, que duró alrededor de seis meses, además de negociar con los agentes sociales del territorio la forma de investigar el problema, se realizó un mapeo de actores involucrados con el cual se logró acercar a los investigadores al equipo de salud del centro asistencial docente de la zona, para dialogar sobre la magnitud y los efectos de la violencia contra las mujeres en el territorio. Este acercamiento generó la formación del grupo motor de personas constituido por cuatro profesionales del centro de salud (trabajadora social, enfermera, matrona y psicóloga), seis dirigentes sociales integrantes del comité de salud (cinco mujeres y un hombre), y cinco integrantes del equipo de investigadores, que acordaron colaborar con la investigación.

Seguidamente, este grupo elaboró una estrategia para realizar un diagnóstico participativo de caracterización del problema en el territorio, delimitando a quiénes se entrevistaría, escogiendo las técnicas de recolección de la información, y elaborando un plan de trabajo para realizar dicho diagnóstico.

### Segundo paso: trabajo de campo y escucha (salir y escuchar a la gente)

En esta etapa del proceso, de catorce meses de duración, se aplicaron distintas técnicas de recolección de información de tipo cualitativo adoptando un enfoque participativo. En síntesis, se conversó con los distintos actores del territorio: pastores evangélicos, profesionales de la salud, mujeres maltratadas, el machi (chamán en la cultura mapuche), la Directora Regional del Servicio Nacional de la Mujer, niños y niñas de las escuelas del territorio, profesores de las escuelas y carabineros utilizando técnicas como entrevistas, grupos de discusión y talleres. Este proceso buscó estimular la participación y la expresión de opiniones de todas las personas vinculadas con el problema, siguiendo los principios metodológicos y éticos de la investigación social. Las conversaciones se grabaron y se obtuvo el consentimiento informado de todos los participantes.

### Tercer paso: la superación del diagnóstico, análisis de la información y devoluciones creativas

El análisis de la información obtenida se realizó mediante la identificación de frases textuales extraídas de las transcripciones de las entrevistas, los grupos de discusión y los talleres. En dicho análisis se hicieron las siguientes lecturas: 1) lectura de fondo: para identificar desde estos discursos los sentidos, representaciones, posiciones y relaciones que los actores le otorgan o tienen con la violencia contra las mujeres, 2) lectura por temas: para conocer los ejes temáticos (categorías) expresados por los distintos actores respecto a este tipo de violencia, y 3) lectura relacional, para señalar los nodos críticos o, dicho de otro modo, los ejes donde convergen los discursos de los entrevistados.

Luego, y de modo participativo, se ordenó e interpretó dicha información, para devolverla sistematizada y generar la lectura crítica del grupo y el consecuente autodiagnóstico participativo, que tuvo como fin afianzar el análisis realizado y validar los sentidos, las posturas y las relaciones de los actores involucrados sobre el problema de la violencia contra las mujeres en el territorio de Boyeco.

### Cuarto paso: organización de la propuesta de abordaje y programación de acciones integrales y sostenibles

Este es el paso en que se encuentra actualmente esta investigación. En él se están fortaleciendo las redes democráticas y participativas que se han generado durante el proceso de IAP en violencia contra las mujeres y el grupo motor es una estructura fundamental. Con esta finalidad, se está desarrollando un proceso transparente de toma de decisiones de abajo hacia arriba en el cual cualquier análisis o propuesta tendrá la oportunidad de ser considerada, debatida y ponderada de modo que el modelo de intervención social para el abordaje de la violencia contra las mujeres que se diseñe, en conjunto, sea pertinente socioculturalmente, eficiente y sostenible.

## ENSEÑANZAS APRENDIDAS Y SIGUIENTES PASOS

Hasta la fecha, se han acumulado enseñanzas significativas y de diversa índole. En resumen, estas enseñanzas incluyen desde la identificación de diversas formas de comprender la violencia contra las mujeres por parte de hombres, mujeres y profesionales del territorio, hasta la desnaturalización de un problema que hasta hace poco tiempo las personas que vivían en el territorio no reconocían como un problema social que les afectara.

Se han identificado, además, cinco elementos críticos que configurarán el problema, de acuerdo con los discursos de las personas entrevistadas. El principal es el machismo presente entre las personas que viven en el territorio, entendido como una forma de relaciones sociales donde predominan ciertas ideas sobre cómo deben ser las relaciones entre hombres y mujeres, y según el cual generalmente se espera que las mujeres se ubiquen en el ámbito de lo doméstico y se encarguen del cuidado de la familia.

Esta creencia se articula con un segundo elemento, el que corresponde a las profundas trasformaciones culturales que viven las familias mapuche del territorio, producto actualmente del avance tecnológico, pero que deviene histórico a partir de la colonización española, primero, y del Estado chileno, después. Esta amalgama de procesos y sus efectos han genera-do una especie de frustración social endémica, que se entrelaza con otros elementos que potenciarían la violencia contra las mujeres: el alto consumo de alcohol por parte de los hombres y el debilitamiento del sustrato social, cultural e identitario mapuche, que regula las relaciones entre quienes viven en el territorio. Por último, la incidencia del papel institucional del Estado en la superación del problema es escasa, porque su ámbito de acción está más orientado a sancionar la violencia que a prevenirla.

A partir de este proceso abierto surgen numerosos interrogantes nuevos, como los siguientes: ¿qué papel están desempeñando las organizaciones sociales del territorio en relación con el problema?, ¿qué elementos culturales habrá que recuperar o fortalecer para iniciar su erradicación?, ¿cuáles serán las aportaciones de los servicios de salud para afrontar el problema de forma intercultural en el caso de la región de La Araucanía?

Por ahora, quienes han participado en el proceso creen que la implementación de esta IAP ha sido una oportunidad para fortalecer los vínculos comunitarios y que se están revalorizando los espacios de diálogo con y entre las personas, la participación social, así como la democracia en la generación de conocimientos pertinentes y participativos de un problema de salud pública que les afecta cotidianamente.

Los siguientes pasos consistirán en diseñar de forma participativa una propuesta de abordaje de la violencia contra las mujeres, con componentes territoriales y culturales, que ayude a articular a las acciones de los actores sociales e institucionales motivados y comprometidos con el abordaje de este problema social urgente, de tal modo que se pueda ayudar a generar políticas sociales pertinentes para el contexto regional de la Araucanía de Chile.

## Agradecimiento.

Los autores agradecen a los habitantes del Lof Boyeco de la región de la Araucanía de Chile su compromiso y motivación para participar en esta propuesta de investigación colectiva.

## Financiación.

Este artículo es un producto del proyecto de investigación FONDECYT 01130542, que ha sido financiado por el Consejo Nacional de Ciencia y Tecnología de Chile (CONICYT).

## Declaración.

Las opiniones expresadas en este manuscrito son responsabilidad del autor y no reflejan necesariamente los criterios ni la política de la *RPSP/PAJPH* y/o de la OPS.
